# Comment on: Fracture risk assessment in patients with type 2 diabetes mellitus

**DOI:** 10.15537/smj.2022.43.9.20220601

**Published:** 2022-09

**Authors:** Ali S. Jawad

**Affiliations:** *Consultant, Department of Rheumatology The Royal London Hospital London, United Kingdom*

*To the Editor*

I have read with interest the study by Alsaedi et al.^1^ Type 1 diabetes mellitus (TIDM) and type 2 diabetes mellitus (T2DM) are associated with increased risk of fractures irrespective of bone mineral density (BMD). There is an increase in fractures at proximal femur, proximal humerus and the ankle with increased postoperative mortality of up to 57% in women and 28% in men.^2^ We agree with Alfadhli et al1 that the fracture risk assessment (FRAX) tool underestimates fracture risk T2DM, more so if bone mineral density (BMD) is included. Though T1DM is considered in Fracture Risk Algorithm (FRAX) under secondary osteoporosis, evidence suggests that fracture risk is also underestimated.^2^

There are other fracture risk assessment tools that are well validated; the United Kingdom (UK) QFracture and the Australian Garvan Fracture Risk calculator.

QFracture incorporates both T1DM and T2DM and hence an alternative to FRAX in assessing fracture risk in patients with diabetes.^3^ QFracture^®^-2016 risk calculator can be accessed on http://qfracture.org. It is recommended for use in England, Scotland, and Wales. It gives a 10-year probability of fracture, and unlike FRAX, it can be used for people between the age of 33 and 99 and it does not include BMD which is an advantage as BMD tends to be higher in patients with T2DM. It does include falls, an important contributor to increased fracture risk in patients with diabetes. There are at least 2 limitations to the use of QFracture in diabetes. It needs direct evaluation in patients with diabetes and calibration in populations outside the UK.

The Garvan Fracture Risk Calculator is another tool and can used.^4^ Five key risk factors for fracture are included: a person’s age, weight, BMD, a history of previous fractures and a history of falls.^5^ Using this information, an algorithm of risk forms the basis of the calculator tool and is useful in patients with diabetes as it includes falls, but again more direct evaluations in patients with T1 & 2DM is required.

More collaborative international research is needed to improve the predictive value of fracture risk assessment tools and the incorporation of both types of diabetes as a risk factor in current and future fracture predictive tools.

## Reply from the Author

No reply was received from the Author.
